# Effectiveness of an innovative community-based breast cancer awareness workshop

**DOI:** 10.1186/1753-6561-6-S4-O25

**Published:** 2012-07-09

**Authors:** A Al-Shammari, A Al-Dahlawi, A Nurhussen, S Obeidat, M AbouSaleh, S Al-Jazairy, MH Rajab

**Affiliations:** 1 Alfaisal University, College of Medicine and Zahra Association for Breast Cancer, Saudi Arabia

## Introduction

Breast cancer is the second leading cause of cancer related death in women. Currently, few women in Saudi Arabia get screened for breast cancer. A recent study found a gap between awareness and the practice of breast self-exam (BSE), and emphasized the need for community-based awareness programs. The main objective of this study was to assess the effectiveness of an innovative community-based breast cancer awareness workshop in increasing the level of confidence in performing BSE in Saudi women.

## Methods

An innovative community-based breast cancer awareness workshop was designed and subsequently conducted in two locations within the City of Riyadh during the months of March and April 2010. The workshop included different techniques that were designed based on neuro-linguistic programming (NLP), which targets three types of learners: visual, auditory, and kinesthetic. A two-part (before and after) questionnaire was used to assess the effectiveness of the workshop in increasing the level of confidence in performing breast self-examination and to measure participants’ satisfaction with workshop content and execution. The study was a non-randomized, pre-post design. Data were analyzed using McNemar test. A p< 0.05 indicated a statistical significance.

## Results

A total of 89 women participated in the two workshops; 74% were of Saudi nationality and the rest were mainly from neighboring Middle Eastern countries. Sixty-six percent of participants were married, 62% were college educated, and 77% were over 26 years of age. At Baseline, 98% of study participants reported that early detection of breast cancer is very important to them as it improves the chances of successful treatment. In relation to breast self exam (BSE), 69 participants (78%) reported knowing how to perform the BSE, out of which 43% reported performing it once per year. At baseline only 15% of participants reported being confident in performing BSE compared to 59% after completing the workshop, (p <0.00). At the end of workshops, 94% of the participants evaluated the workshop as being excellent and 91% reported that the workshop met their expectations.

## Conclusions

Based on the survey results, the community-based awareness workshop employed was effective in increasing the level of reported confidence in performing BSE among the study participants.

